# Procalcitonin: Infection or Maybe Something More? Noninfectious Causes of Increased Serum Procalcitonin Concentration: Updated Knowledge

**DOI:** 10.3390/life15030446

**Published:** 2025-03-12

**Authors:** Szymon Mućka, Grzegorz K. Jakubiak, Natalia Pawlas

**Affiliations:** Department of Pharmacology, Faculty of Medical Sciences in Zabrze, Medical University of Silesia, Jordana 38 St., 41-800 Zabrze, Poland; mucka.szymon@gmail.com (S.M.); grzegorz.k.jakubiak@gmail.com (G.K.J.)

**Keywords:** procalcitonin, circulating biomarkers, inflammation, carcinoma, autoimmunological diseases, hepatotoxicity, renal failure, injury, surgery

## Abstract

Procalcitonin (PCT) is a precursor of calcitonin, and its determination is used in daily clinical practice. It is a good marker for bacterial infection and can help diagnose sepsis. In this review, we summarize recent findings on the utility of PCT serum concentration measurement in noninfectious conditions. We found that elevated PCT levels may help in diagnosing or monitoring the course of cancer or inflammatory diseases. An increase was observed in emergency care such as acute renal failure or injuries, which may be promising in estimating the risk of complications. PCT has the potential to become a useful and clinically relevant marker beyond the assessment of bacterial infection. Due to its limited specificity, therapeutic decisions should be based on an individual evaluation of each clinical case.

## 1. Introduction

Procalcitonin (PCT) is a protein composed of 116 amino-acids, and the encoding gene is located on chromosome 11. PCT is a precursor for calcitonin, which is produced in the parathyroid cells of the thyroid gland. Translational activity is mostly detectable in thyroid and lung neuroendocrine cells [1,2]. PCT, as a result of cleavage, becomes calcitonin, an important hormone responsible for calcium homeostasis in the bloodstream. One molecule of PCT is converted into one molecule of calcitonin. Importantly, the dissociation process occurs only inside the cell. Because of this, PCT is found in negligible concentrations in the blood in a physiological state. Its levels in healthy people are less than 0.1 ng/mL [3]. Currently, a common and effective method for detecting PCT concentrations in serum is the immunoanalytical method. However, there are new methods constantly being tested with promising values for sensitivity and specificity [4,5]. Determining the half-life of PCT is not simple. It is known that the process is dependent on the glomerular filtration rate, and in patients suffering from chronic kidney disease, elevated levels can persist for a long time. It is now accepted that PCT can persist in plasma for 24 h, after which the concentration decreases [6,7].

PCT was first described by Moya et al. in 1975 [8]. Over a dozen years later, Assicot et al. made a breakthrough discovery. They discovered that PCT levels increase in patients during sepsis [9]. Since then, there have been many recommendations for antimicrobial treatment, with the Surviving Sepsis Campaign guidelines leading the way. The researchers described how to interpret PCT levels and how to use the results in practice. Due to the demonstrated lack of benefit of serum PCT determination in randomized clinical trials (RCTs), it is suggested (due to weak recommendation and extremely low quality of evidence) that this test not be performed as part of standard clinical evaluation [10].

However, in clinical practice, physicians encounter situations where the clinical picture may suggest the presence of an infection of unknown etiology. This results in implementing empirical antibiotic therapy, which turns out to be unnecessary or too long. Elevated PCT levels can prove to be a valuable tool for estimating the risk of bacterial infection, which can reduce the unnecessary use of antibiotics. This is extremely important because it reduces the selection of resistant strains, which is important for public health [11]. Similarly, this was the problem faced by many practitioners during the COVID-19 pandemic. The main problem with determining the usefulness of PCT was differentiating viral-only infections versus additional bacterial infections. PCT did not directly predict what the prognosis was, but it was a valuable guide for physicians to make individual therapeutic decisions. Other well-known and commonly used inflammatory parameters include C-reactive protein (CRP), lactate dehydrogenase (LDH), the white blood cell count, and interleukin-6 (IL-6) [12,13]. Of course, it should be noted that the cause of a PCT increase may be an accompanying infection, but studies have shown an independent increase in PCT.

The last such study summarizing noninfectious causes of an increase in the PCT serum concentration was released more than 10 years ago, and an update is needed [14]. We searched the PubMed database for relevant works. To find the bibliography, we used the following commands: “procalcitonin”, with the filters applied being in the last 5 years, Adaptive Clinical Trial, Classical Article, Clinical Study, Clinical Trial, Clinical Trial Protocol, Comparative Study, Controlled Clinical Trial, Guideline, Meta-Analysis, Multicenter Study, Observational Study, Practice Guideline, Pragmatic Clinical Trial, Randomized Controlled Trial, Review, Systematic Review, English, and Humans. We then rejected articles assessing the role of PCT in infections. In cases of insufficient research, we extended the search to 10 years back. We analyzed 74 studies which met the above inclusion and exclusion criteria.

The purpose of this paper is to summarize the disease states associated with elevated PCT levels as well as the clinical utility of PCT measurements in such patients. In this review, we emphasize the most important findings of recent years, with particular attention paid to studies where PCT levels remained elevated despite the exclusion of infection. In this paper, we not only mention clinical situations but specify which values are clinically relevant, depending on the disease.

## 2. Noninfectious Causes of Increased Serum Procalcitonin Concentration

### 2.1. Cancer Causes

Tumors, especially neuroendocrine neoplasms, can release molecules into the bloodstream. Many of these are valuable markers of certain cancers. Concerning PCT, there is still a lack of clear guidelines on clinical utility [15]. The researchers found elevated levels of inflammatory parameters, including PCT, in the terminal stage of most common cancers. An increase in this parameter correlated with higher mortality, but of crucial importance is that it was independent of concomitant infection [16]. Below, we describe the most common tumor types in which the increase in PCT was significant.

#### 2.1.1. Medullary Thyroid Carcinoma

Medullary thyroid carcinoma (MTC) is a relatively rare type of cancer for this organ. Underlying the neoplasm is a neoplastic transformation of the perihilar cells of the thyroid gland, which produce calcitonin. Specifically, this marker is currently the standardized and sufficiently sensitive marker of this type of neoplasm. Cancer cells also release increased amounts of the calcitonin precursor into the bloodstream, particularly in calcium-stimulated tests, which triggers a cascade and results in an increase in PCT and then calcitonin [17,18,19]. Can the calcitonin precursor also be an indicator of MTC?

Cases of accidental diagnosis of cancer have been reported more than once. Patients were treated for another reason, but their PCT levels remained above the upper limit of what is considered normal, even though their symptoms had resolved [20,21,22,23,24]. The PCT concentration may increase in MTC because tumor cells release it into the bloodstream [3]. Giovanella et al. indicated in their meta-analysis that PCT may be a valuable and promising biomarker of cancer in patients with previously diagnosed nodular thyroid disease [25]. Some researchers determined in their work that PCT shows high sensitivity and specificity in detecting cancer [26,27]. In another retrospective study, it was shown that PCT increases significantly only in MTC. In follicular, papillary, and anaplastic carcinoma, no significant increase in this biomarker was observed [28]. However, caution should be exercised when therapeutic decisions are based on elevated PCT levels alone. To increase the reliability of diagnosis, it is recommended to test for calcitonin and PCT simultaneously [18,26,29].

There are still no clear guidelines for the lowest concentration value which could be diagnostically relevant. Some researchers suggested a cut-off threshold of 0.1 ng/mL [19,27,29,30], while other researchers used a lower cut-off value of 0.07 ng/mL [26]. The proposed values for the standard are given in Table 1. Difficulties in setting diagnostic values may be due to wide individual variability due to factors such as gender [30]. At this point, it is worth remembering that a concentration below 0.05 ng/dL is considered normal.

Another major problem is the diagnosis of a tumor according to its size. For small MTC tumors, PCT remained non-detectable. Moreover, tumor size was expressed poorly as an increase in PCT concentrations [26].

More studies should be carried out, especially with patients in whom PCT persists despite the exclusion of infection. In addition, tests comparing calcitonin and PCT in the diagnosis of medullary thyroid cancer should be performed. Evaluation tests for treatment monitoring and prognosis estimation should be considered.

#### 2.1.2. Small-Cell Lung Cancer

Small-cell lung cancer (SCLC) is a neuroendocrine tumor of the lung strongly correlated with cigarette smoking. Neoplastic cells can secrete biologically active substances, leading to the manifestation of non-specific systemic symptoms and paraneoplastic syndromes [31]. Researchers demonstrated that SCLC can produce PCT. The release of PCT can be used as a marker for this tumor, which may be useful in diagnosing or monitoring the course of the disease [32,33]. Stando and Chmielewski described a case with extremely high values for this marker, while the levels of other inflammatory parameters were in the normal range. Increasing PCT levels correlated with tumor progression, reaching triple-digit numbers [34]. Chen et al. showed that PCT might also be useful for determining prognosis, as higher PCT levels correlated with worse treatment responses. The cut-off point was defined as >0.06 ng/mL [35].

#### 2.1.3. Non-Small-Cell Lung Cancer

Generally, no increase in PCT was observed in this type of cancer [33]. Another study showed that PCT levels may be elevated in non-small-cell lung cancer. What is more, it was proven to be a poor prognostic marker and an indication of deterioration in the quality of life in patients [36]. Due to inconclusive scientific reports, the biomarker’s contribution to this type of cancer should be further explored.

### 2.2. Hypercalcemia

The state of elevated serum calcium triggers the secretion of calcitonin, which is essential in maintaining balance in calcium metabolism. Provocative tests during which patients took exogenous calcium were mentioned earlier. Such stimulation results in an increase in calcitonin production from PCT. Such a test has also been shown to result in the increased release of PCT into the bloodstream. Other studies reported that tumor hypercalcemia stimulates calcitonin to secrete. There is a lack of conclusive studies indicating an increase in PCT due to tumor or metabolic hypercalcemia. New research should focus on assessing the impact of hypercalcemia on PCT levels in various diseases, particularly cancer and electrolyte imbalances [17,26,37].

### 2.3. Autoimmune Diseases and Immunological Reactions

Autoimmune diseases are conditions where there is an excessive inflammatory response, resulting in symptoms. Their heterogeneity results in different constellations of signs and markers [38].

Systemic lupus erythematosus (SLE) is a chronic autoimmune disease characterized by a complex pathomechanism. A crucial role is played by abnormal immune responses, which lead to the formation of immune complexes and organ damage. Markers such as ANA, anti-dsDNA, anti-Sm, and antiphospholipid antibodies are essential in the diagnosis of this disease. The guidelines have no direct reference to PCT as a biomarker in the diagnosis or monitoring of this disease. Certain subtypes of inflammatory or autoimmune diseases can release proinflammatory markers into the blood, such as TNF-alpha or interleukin-6. These in turn can affect the secretion of PCT by cells [39]. Researchers indicated that PCT values may be elevated in chronic lupus patients with an associated infection. Although PCT was significantly increased in the group with infections, PCT was nonnegative in the no-infection group. The values were 1.1 ± 0.2 for the group with infections and 0.7 ± 0.3 for the group without infections [40]. Similarly, Aydin and Turk showed higher PCT levels in patients hospitalized in an intensive care unit for exacerbation of rheumatic diseases, with significantly higher values in the patients who died [41]. In contrast, in the juvenile variant of the disease, patients had no significantly noticeable concentration of this parameter. However, a larger group should be studied to increase reliability [42]. Aringer’s review points out the possible elevation of PCT in patients diagnosed with SLE, but these values are not sufficient to follow the course and severity of the disease [43]. A moderate increase in this biomarker was also observed in rheumatoid arthritis [44].

Inflammatory diseases in children can present similar symptoms and be a challenge for experienced pediatricians. In Kawasaki disease, a high increase in inflammatory parameters can be observed [45]. However, in a meta-analysis, Pan and Fan showed that PCT has low predictive value in this disease and should not be used [46]. Quite different is the case of multisystem inflammatory syndrome. In this disease, a large increase in PCT has been observed, which can be an important tool for diagnosing this disorder or differentiating it from Kawasaki disease [47,48].

Of interest is a case report in which there was a significant increase in PCT after vaccination against COVID-19 [49]. It has previously been reported that PCT increased in children with vaccine reactions. More research needs to be conducted, especially to determine the risk of serious infection, to avoid unnecessary antibiotic use [50].

### 2.4. Liver Failure

The most common cause of acute liver failure (ALF) in developed countries remains paracetamol overdose. Timely initiation of detoxification therapy and vigilant monitoring for signs of liver failure are essential strategies in reducing mortality rates associated with paracetamol poisoning. Assessment of liver damage can be performed by measuring serum transaminase activity or prothrombin time, as well as advanced molecular methods [51,52]. Many researchers reported that PCT is a potential marker of liver failure. Furthermore, the increase in PCT levels was earlier and more dynamic than that of aminotransferase [53,54]. Another prospective study found that PCT can be a valuable tool for the rapid assessment of ALF and also for implementing appropriate treatment. Importantly, the researchers found that PCT reached extremely high values, being as high as 21.5 ng/mL (3.0–45.3), in the group which developed ALF [55]. A significant discovery was made by scientists led by Zheng. Specifically, they discovered that the source of PCT secretion during ALF is liver monocytes responding to cytokines [56].

Haselwanter was able to prove that the PCT level was elevated in patients with acute-on-chronic liver failure (ACLF). His innovative method reduced the levels of inflammatory mediators, and consequently, there was a decrease in the PCT levels. This suggests an important role for PCT in assessing the development of inflammation in an organism [57]. Igna et al. found that PCT is a useful marker for assessing mortality in patients with liver failure associated with alcoholic cirrhosis. However, it should be mentioned that systemic inflammatory infection was the main mechanism causing mortality [58]. As shown in another study, the level of PCT was elevated after hepatectomy for hepatocellular carcinoma and was a predictor of a worse prognosis. In addition, several of the patients did not develop infections, despite the increased PCT levels [59,60].

### 2.5. Pulmonary Diseases

The second most common disease with elevated PCT, aside from pneumonia, is chronic obstructive pulmonary disease (COPD). Exacerbations of chronic obstructive pulmonary disease most often occur as a consequence of upper respiratory system infections. The onset of such a condition often requires the involvement of additional interventions, as if left untreated, it can lead to death. It is desirable to use medications, including antibiotics, appropriately [61]. Bacterial exacerbation can be indicated by an elevation in PCT levels [62]. It should be remembered that not all exacerbations are caused by infection, and an increase in PCT may be non-specific. Zheng et al. found that PCT levels were elevated in all patients with COPD. Additional conditions or abnormalities in lung imaging correlated with higher PCT levels [63]. In patients diagnosed with COPD, PCT reached higher values compared with healthy non-smoking volunteers. It is worth mentioning that saliva concentrations were correlated with serum concentrations [64]. Another study found a worse prognosis in patients hospitalized for COPD exacerbation who had higher levels of PCT. The cut-off point was 0.25 ng/mL [65]. Similarly, in asthma, PCT has been found to be useful in identifying the causes of symptom exacerbations. With this biomarker, it is possible to distinguish between exacerbations of viral and bacterial etiologies. This helps reduce the usage of antibiotics and estimate prognoses [66].

Researchers have found interesting results regarding smoking. Rudzinska-Radecka et al. found that in smokers without COPD, the average PCT concentrations were significantly higher compared with healthy volunteers without cigarette addictions [64].

### 2.6. Renal Failure

As mentioned in the introduction, the excretion of PCT occurs predominantly in the renal system. It is worth mentioning here that renal clearance of PCT is not linear and largely depends on the patient’s renal function. In patients with renal failure, it can extend for up to 44 h at a filtration rate of <30 mL/min, while in healthy individuals, PCT is cleared in the range of 25.2–30.0 h [6]. Predicting acute kidney injury (AKI) with PCT is difficult in cases of sepsis and current bacterial infection. Moreover, an increase in the level of the marker may be the result of impaired glomerular filtration because PCT is excreted by the kidneys. However, PCT may be a useful marker for predicting AKI, as a meta-analysis determined its utility with a sensitivity of 0.76 and specificity of 0.75 [67]. In contrast, the study by Shen et al. demonstrated that more dynamic changes in PCT levels can be useful in estimating the prognosis and progression of AKI into the persistent stage. Of note, it was shown that the difference in concentrations in the two blood collections was prognostically significant. In contrast, a single measurement of PCT was found to be of little value in predicting persistent AKI [68].

It has been established that dialysis therapy in chronic kidney disease (CKD) is a pro-inflammatory factor and contributes to increased levels of inflammatory markers [69]. PCT levels were found to be elevated in dialysis patients with chronic renal failure, even though infection was excluded. Moreover, significantly higher values were found in the male group, but no differences were shown between different ages or causes of chronic renal failure. In the entire group of dialysis patients without infection, the mean PCT concentration was 0.50 ± 0.49 ng/mL (*p* = 0.006) [70]. In a later study, the researchers found that PCT was also elevated in CKD and that the concentrations increased as the disease advanced and progressed [71]. In turn, Mouche et al. proved that PCT levels were significantly elevated in dialysis children without signs of infection. In these patients, dialysis caused a rapid decrease in the concentration of this biomarker. The researchers pointed out that high concentrations or a dynamic increase should be interpreted as a suspicion of infection rather than a worsening of glomerular filtration [72].

Given this, future studies should consider the renal mechanism of PCT excretion. In renal failure, PCT may sustain higher values, regardless of a coexisting infection or other condition releasing inflammatory markers.

### 2.7. Cardiovascular Diseases

PCT is an important marker in determining cardiovascular risk, especially in patients with metabolic diseases. In a cross-sectional study, Katte et al. showed a statistically significant increase in this biomarker in patients with diabetes. Interestingly, higher concentrations of PCT were found in women than in men, and the marker was found to be more predictive of cardiovascular disease than CRP [73]. In another study, PCT appears to be a valuable tool in differentiating the causes of dyspnea. With low PCT levels, it is possible to exclude pulmonary infection and focus on treating the exacerbation of heart failure. It is worth mentioning that PCT is not a reliable diagnostic parameter in heart failure like traditional markers (e.g., B-type natriuretic peptide or N-terminal pro-B-type natriuretic peptide). On the other hand, PCT can be a valuable predictor of impaired prognosis, especially in combination with other markers [74,75,76,77]. Similarly, in another study, heart failure can cause elevated PCT levels and can be indicative of a worse prognosis. Extreme caution should be exercised in making therapeutic decisions based on the result of this biomarker, especially in patients with possible coexisting pulmonary infection [14].

There is a study which assessed the value of PCT in cardiac ischemia. In a study evaluating the effectiveness of various biomarkers in distinguishing type 2 myocardial infarction (T2MI) from other types of myocardial infarctions (MIs) and predicting patient outcomes, there were elevated levels of PCT. This was associated with a worse prognosis in patients with T2MI, and this suggests that PCT may be a useful prognostic marker in this group of patients [78].

In another study, researchers focused on the risk factors associated with heat stroke, including intracranial hemorrhage. PCT was identified as one of the biomarkers associated with the severity of the condition. The mean value in all patients was 1.745 (0.71–3.84) ng/mL, and in patients complicated by hemorrhages, it was 4.75 (1.7–18.67) ng/mL. Elevated PCT levels were associated with a higher risk of complications and a worse prognosis [79].

In conclusion, PCT can be used as a prognostic indicator in cardiovascular disease, but the general clinical condition of the patient should be taken into consideration, and the results should be interpreted individually.

### 2.8. Injuries

Sinha et al. found that the mean PCT value was elevated in patients admitted due to burns independent of the following prognosis. However, the values of this marker were higher in the patients who passed away. It has been suggested that values above 2.1 ng/mL are an indicator of an inauspicious prognosis and require special care. It is interesting to note that the PCT values already reached high values on the first day after an accident [80]. Similarly, other researchers reported in their meta-analysis that the PCT levels were significantly increased and correlated with a more frequent mortality rate [81].

This was no different in a study evaluating multi-organ injuries, excluding burns. These injuries were an independent factor in the massive elevation of PCT levels in children treated for this reason in an intensive care unit. The average value in all patients on admission was nearly more than 4 ng/mL. Astonishingly, in those who later died, the level was over 20 ng/mL [82].

Another study tried to determine if PCT could be a useful marker in determining the severity and prognosis of hemorrhagic shock. It appeared that PCT was not a useful tool for differentiating between the septic and hemorrhagic etiologies of shock [83].

### 2.9. Surgeries

Surgery is associated with an inflammatory response, resulting in the release of various mediators into the bloodstream. Depending on the extent of the procedure and the location, the intensity of the release can be varied and heterogeneous [84]. PCT levels increase in cardiac surgery procedures, peaking on the first postoperative day, which indicates an inflammatory response to surgery [85]. Another study found that this marker can be helpful for excluding intestinal ischemia after vascular surgery. This has particular prognostic significance for patients [86]. Analysis of the change in neonatal PCT concentrations was performed according to mode of delivery. PCT values were significantly elevated during the first 48–72 h after both modes of delivery [87]. However, a cesarean section may raise PCT levels, and thus it is suggested that this biomarker is useful in predicting infection [88]. Researchers have shown that an increase in inflammatory mediators is a response to cardiopulmonary bypass surgery without the features of a previous infection. Remarkably, the increase in PCT was observed after 24 h and occurred later than some inflammatory mediators, which may indicate a cascade of mediator release. Furthermore, the use of a steroid resulted in a reduced inflammatory response, which directly contributed to less PCT released into the bloodstream [89]. Another study on the same procedure showed that PCT levels were also higher already on the first day, with values oscillating near 3.24 ± 8.48 ng/mL in all patients [90].

Researchers have more than once pointed to multifactorial causes of increased PCT. Surgery by itself may cause an increased release of inflammatory mediators or contribute to oxidative stress, especially in procedures complicated by local ischemia. Finally, it should be remembered that a disruption in skin integrity may be associated with bacterial invasion, and infection is an acknowledged factor in the growth of PCT. The above cases should be considered on a case-by-case basis, especially when a surgical operation may be accompanied by an infection.

### 2.10. Pregnancy and Delivery

A study was carried out to determine if a healthy pregnancy could be the cause of elevated PCT levels. Joyce et al. demonstrated that in normal pregnant women in the third trimester, PCT values remained decreased and did not exceed 0.25 ng/dL. Furthermore, PCT did not increase directly after delivery [88]. Researchers led by Dockree came to similar conclusions. In this study, healthy women also had no elevated PCT values and were comparable to healthy people not experiencing pregnancy (less than 0.05 ng/dL) [91]. PCT also did not differentiate between preterm and term deliveries. In both pre- and post-37-week births, PCT values were sustained below 0.05 ng/dL [92]. In the diagnosis of obstetric diseases, PCT is of limited importance. In a study evaluating the usefulness of serum markers, it was shown that PCT is not significant in detecting asphyxia in a term fetus [93]. A meta-analysis by Areia showed that PCT is a poor factor in detecting chorioamnionitis and is not more valuable than CRP. It was therefore recognized that PCT is an important parameter which can indicate severe infection or sepsis in pregnancy or after delivery, which can be valuable in the rapid initiation of antimicrobial treatment [94]. The use of PCT should be considered if bacterial infection or coexisting sepsis is suspected. Such management can reduce the unnecessary use of antibiotics [91]. At the same time, its use is severely limited in the preterm rupture of membranes. Moreover, PCT should not be used instead of well-established inflammatory parameters due to its relatively low sensitivity and specificity values [95].

## 3. Conclusions

Elevation of PCT can occur in conditions other than bacterial infection. It is worth considering developing standards for diagnosis and treatment follow-up based on the marker. The reliability of the marker increases when it is combined with general clinical assessment and other inflammatory mediators. The PCT value is particularly important for estimating whether a clinical case is complicated by a bacterial infection. When PCT values remain elevated despite the absence of infection, the physician should remain vigilant and, if possible, extend the diagnosis according to the patient’s clinical condition. The main limitation of PCT is its low specificity, which can make it difficult to make a definitive diagnosis. Particularly problematic are clinical situations in which the infection is subclinical or undetectable by standard methods and the PCT is still elevated. In addition, improved detection techniques increase the availability of this test. Future studies should focus on analyzing inflammatory mechanisms, especially those which are associated with cancers, autoimmune diseases, and events with skin disruption. This review did not accurately explain all diseases which are associated with higher levels of PCT. Research is certainly needed, especially for conditions in which PCT is elevated in a moderate range or of uncertain etiology. Oncology and autoimmune disease research require special attention. It is in these areas that the multifaceted and complex etiology of PCT secretion is observed. This will allow a deeper understanding of the pathomechanisms of the release of this biomarker.

## Figures and Tables

**Table 1:** Comparison of cut-off point, sensitivity, and specificity for procalcitonin (PCT) in medullary thyroid cancer.

Cut-Off Point [ng/dL]	Sensitivity/Specificity	Reference
0.07	85.7%/98.9%	[26]
0.1	100%/90%	[27]

## Data Availability

Not applicable.
